# Infrared thermography-based radiomics for early detection of metabolic syndrome

**DOI:** 10.1038/s41598-025-98831-1

**Published:** 2025-04-22

**Authors:** Jiayang Guo, Huizhong Xue, Yu Chen, Xiaoran Li, Yiyun Chen, Xianhui Zhang, Yanhong An, Hua Zhang, Yimeng Yang, Luqi Cai, Wenzheng Zhang, Yonghua Xiao

**Affiliations:** 1 The First Clinical Medical School, Beijing University of Chinese Medicine, Beijing, China; 2 Dongzhimen Hospital, Beijing University of Chinese Medicine, Beijing, China

**Keywords:** Infrared thermography, Metabolic syndrome, Radiomics, Machine learning, SHAP, Metabolic syndrome, Imaging and sensing, Diagnostic markers

## Abstract

Radiomics is increasingly utilized in medical image analysis. This study evaluates the use of infrared thermography, a technique well-suited for radiomic analysis, in diagnosing metabolic syndrome (MS). Facial and palmar thermographs from 200 males (100 healthy controls and 100 MS patients) were analyzed. The dataset was split into a training cohort (*n* = 140) and a validation cohort (*n* = 60). All participants underwent laboratory testing on the same day as infrared thermography imaging. A total of 1656 radiomic features were extracted from each participant’s thermographs and refined using Pearson correlation coefficients, two-sample t-tests, and LASSO regression. A binary random forest (RF) classification model was then constructed and evaluated based on its calibration, discrimination, and clinical utility. The RF model demonstrated strong diagnostic performance, achieving an AUC of 0.91 in the validation cohort. Calibration and decision curve analyses confirmed the model’s clinical applicability. Infrared thermography-based radiomics offers a promising, non-invasive method for early screening of MS, highlighting its potential clinical utility.

## Introduction

Radiomics, an emerging field in medical imaging, employs high-throughput algorithms to extract quantitative features from clinical images, enabling the detection of subtle patterns beyond human visual perception. Initially applied to computed tomography (CT) in oncology^1^, radiomics has expanded to magnetic resonance imaging (MRI), positron emission tomography (PET), and ultrasound, demonstrating broad utility in disease diagnosis, prognosis prediction, and treatment monitoring^2,3^. However, its application to functional imaging modalities, such as infrared thermography (IRT), remains underexplored.

**Fig. 1:**
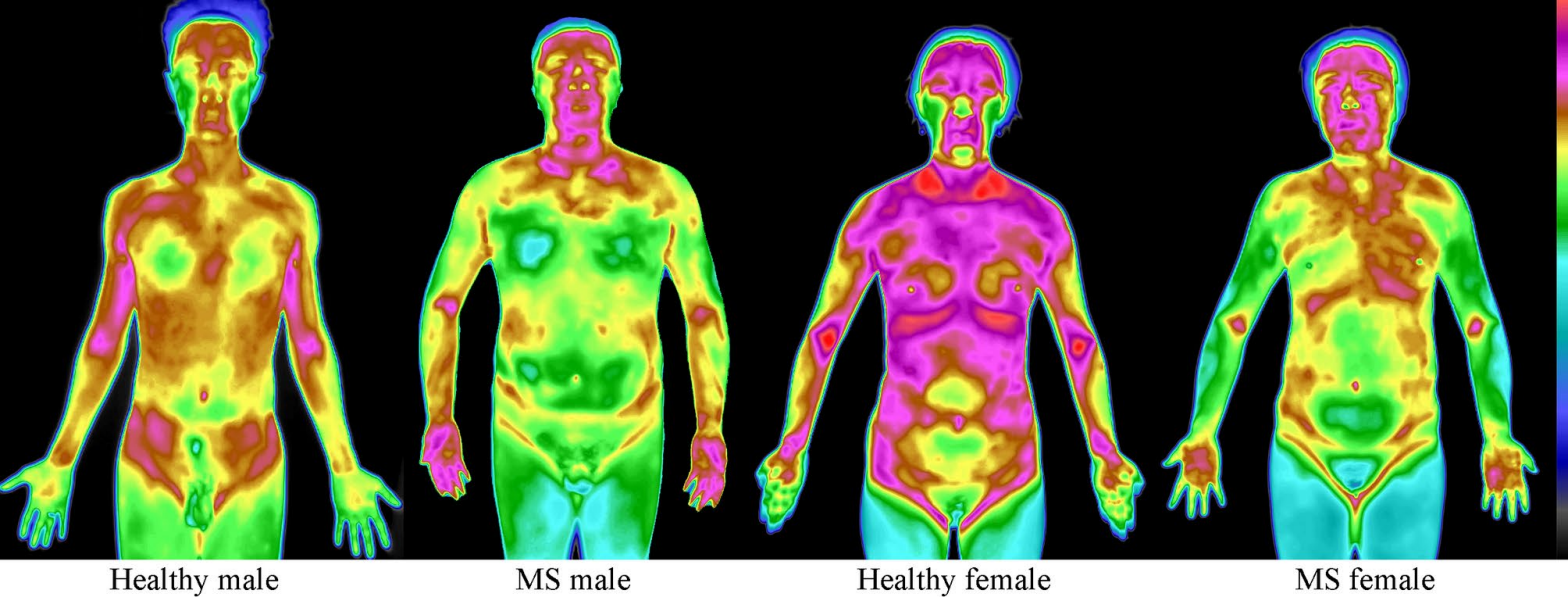
Overall heat distribution patterns of healthy individuals and participants with MS. The infrared thermogram reveals distinct thermal patterns between the two groups. Compared to healthy individuals, those with metabolic syndrome display relatively elevated temperatures in the face and palms. All thermograms were analyzed with standardized parameters: window width 12 °C, window level 31 °C, and display range (25–37 °C).

IRT captures surface temperature distribution by detecting infrared radiation emitted from the human body. According to Pennes’ bio-heat equation, skin temperature reflects localized blood perfusion and metabolic activity^4^, making IRT a promising tool for assessing metabolic disorders. For instance, chronic inflammation and insulin resistance—hallmarks of metabolic syndrome (MS)—alter vascular function and heat production^5–8^, potentially manifesting as distinct thermal patterns on facial and palmar regions (Fig. 1). While traditional IRT studies focused on localized temperature changes in conditions like peripheral vascular disease^9–13^, recent advances highlight the diagnostic value of global thermal distribution in metabolic disorders such as diabetes and hypertension^14–17^.

Despite its potential, IRT faces limitations in clinical adoption. Current analyses rely on simplistic metrics (e.g., mean temperature), neglecting the high-dimensional texture and statistical features embedded in thermographs. Furthermore, the lack of standardized radiomics frameworks for IRT hinders the translation of thermal data into actionable biomarkers. To address these gaps, we propose infrared thermography-based radiomics, integrating radiomics methodologies with IRT to enhance diagnostic precision for MS.

## Materials and methods

### Study design and participants

This retrospective cohort study received ethical approval from the Ethics Committee of Dongzhimen Hospital, Beijing University of Chinese Medicine (Approval No. 2024DZMEC-130-01), with a waiver of informed consent granted for the use of anonymized historical data. Data were retrospectively collected from individuals who underwent routine health examinations between July 2017 and April 2023. Infrared thermography was included as part of the standard health assessment protocol for most participants, allowing for secondary analysis of anonymized data without the need for additional recruitment. All participants underwent laboratory testing on the same day as infrared thermography imaging. All study procedures adhered to the ethical principles outlined in the Declaration of Helsinki.

We stratified participants into two groups based on established criteria. The metabolic syndrome (MS) group consisted of male participants aged 18 to 80 years who met at least three of the diagnostic criteria for metabolic syndrome according to the Chinese Diabetes Society (2020)^18^. These criteria included abdominal obesity (waist circumference ≥ 90 cm), hyperglycemia (fasting plasma glucose ≥ 6.1 mmol/L and/or 2- h postprandial glucose ≥ 7.8 mmol/L following a 75 g oral glucose tolerance test, or a confirmed diagnosis of diabetes), hypertension (blood pressure ≥ 130/85 mmHg or a confirmed diagnosis of hypertension), elevated triglycerides (fasting triglycerides ≥ 1.70 mmol/L), and reduced HDL-C (fasting HDL-C < 1.04 mmol/L).

The Control group consisted of age-matched male participants who did not meet any of the metabolic syndrome diagnostic criteria.

Exclusion criteria were applied to ensure participant eligibility and included individuals with clinically significant comorbidities, such as coronary artery disease or diabetic ketoacidosis. Additionally, participants with conditions impairing thermoregulatory function, such as thyroid dysfunction or acute infectious diseases, were excluded. Participants with missing essential clinical parameters or infrared thermography data were also excluded from the study.

**Fig. 2:**
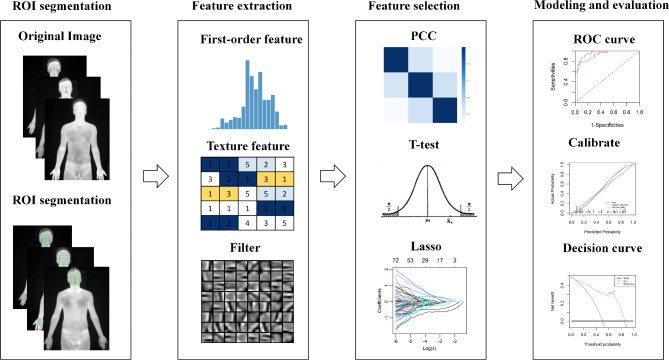
Workflow of infrared thermography-based radiomics analysis.

The workflow of the infrared thermography-based radiomics analysis is illustrated in Fig. 2.

### Thermograph acquisition and ROI segmentation

Thermal imaging was performed using the HIR-2000 A medical infrared camera (Beijing Yuetian Optoelectronics Technology Co., Ltd), which operates within the spectral range of 8–14 μm and has a resolution of 256 × 324 pixels. The camera’s thermal sensitivity is 0.05 °C, and it captures images at a frame rate of 9 fps.

To standardize the imaging process, we controlled the environmental conditions, maintaining a temperature of 22.0 °C ± 2.0 °C and a relative humidity of 60–70%. Prior to imaging, participants were instructed to abstain from alcohol, caffeine, exercise, and exposure to extreme temperatures for at least 4 h. During the imaging procedure, participants were positioned 2.0 m from the camera, which was held perpendicular to the regions of interest (ROIs). The facial (full face) and palmar (right palm) regions were selected as ROIs for thermal imaging. The right palm was chosen due to its anatomical symmetry, while the facial region was selected for its ability to provide insights into systemic metabolic activity, owing to its rich superficial vasculature. Additionally, radiomics features must remain consistent across acquisition, analysis, and quality control processes to ensure their clinical applicability. Standardization of procedures is essential to fully exploit the potential of infrared thermography-based radiomics^19^, Thermal images were processed using 3D Slicer (v5.6.2), in which ROIs were manually delineated by two trained radiologists.

### Feature extraction

In this study, a total of 1656 radiomic features were extracted from palmar and facial infrared thermographs using Pyradiomics v3.0.1. These features were categorized into three primary groups: first-order statistics, texture features, and filter-enhanced features. First-order statistics include intensity distributions, including metrics such as mean, median, skewness, and kurtosis. Texture features were extracted from five distinct feature matrices: the gray-level co-occurrence matrix (GLCM), the gray-level run-length matrix (GLRLM), the gray-level size-zone matrix (GLSZM), the neighboring gray-tone difference matrix (NGTDM), and the gray-level dependence matrix (GLDM). The filter-enhanced features were obtained through multi-scale image transformations, which included Laplacian of Gaussian (LoG) filtering at scales of σ = 0.5, 1.0, and 1.5 mm to enhance fine texture variations, Local Binary Pattern 2D (LBP2D) for microstructural pattern recognition, and wavelet decomposition (‘rbio1.1’ basis) for multi-resolution analysis across frequency domains.

Unlike structural imaging modalities such as CT and MRI, which are constrained by anatomically defined regions of interest (ROIs), infrared thermography lacks standardized morphological references. As a result, shape features were excluded to mitigate segmentation-dependent biases, with emphasis instead placed on intensity- and texture-based biomarkers.

This multi-filter approach enhanced the detection of metabolic syndrome (MS)-associated thermal signatures, resulting in improved pattern discrimination compared to single-modality analyses.

### Feature selection and model development

To reduce dimensionality and improve model performance, we applied a multi-step feature selection process. First, we removed highly correlated features, excluding those with a Pearson correlation coefficient (PCC) greater than 0.99. Next, univariate screening was performed using a two-sample t-test with a significance threshold of *p* < 0.05. Finally, a multivariate selection approach was applied using Least Absolute Shrinkage and Selection Operator (LASSO) regression with 10-fold cross-validation.

For model development, a Random Forest classifier was trained with data from 140 participants (70 metabolic Syndrome (MS) patients and 70 healthy controls). The validation set consisted of 60 participants (30 MS patients and 30 controls). The model was trained with 100 trees, using the Gini impurity criterion and a maximum depth of 5.

### Model evaluation and interpretability

The performance of the model was evaluated using multiple metrics, including Area Under the Curve (AUC), accuracy, sensitivity, and specificity. Model calibration was assessed using Spiegelhalter’s z-test, while Decision Curve Analysis (DCA) was employed to evaluate its clinical utility. For interpretability, we used SHAP (Shapley Additive Explanations) values to rank the importance of features in the model.

## Results

### Participant characteristics

This study included 200 male participants, consisting of 100 patients with metabolic syndrome (MS) and 100 healthy controls, aged 23–76 years. Stratified random sampling was used to allocate 140 participants (70 MS patients and 70 healthy controls) to the training cohort, and the remaining 60 participants (30 MS patients and 30 healthy controls) were assigned to the validation cohort. No statistically significant differences were observed between the health control group and MS group in terms of age and height.

### Radiomic feature selection

**Fig. 3:**
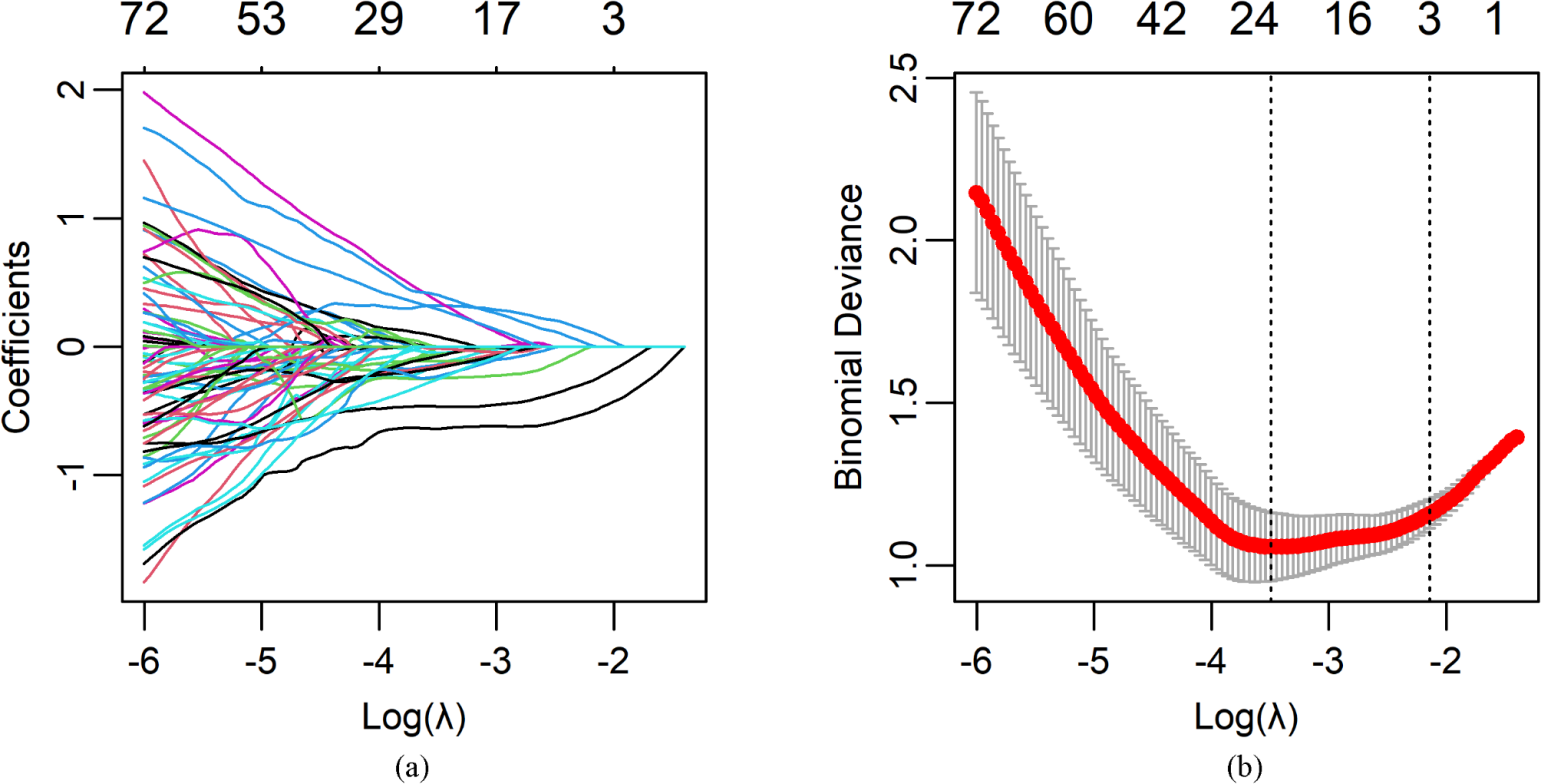
Radiomics feature selection using the Least Absolute Shrinkage and Selection Operator (LASSO) binary logistic regression model. (**a**) Lasso Coefficient Path: This plot illustrates the progression of LASSO coefficients as the regularization parameter (λ) increases. As λ becomes larger, the coefficients of less significant features are progressively shrunk towards zero, effectively performing feature selection. (**b**) Lasso Cross-Validation Curve: This curve displays the cross-validation error as a function of λ. The optimal λ is identified at the point where the cross-validation error is minimized (right dashed line). A feature set is selected based on one standard error from the minimum λ (left dashed line), ensuring a balance between model generalization and the prevention of overfitting.

To identify the significant features associated with metabolic syndrome (MS), we employed several methods, including Pearson correlation coefficient (PCC), two-sample t-test (with a significance level of *p* < 0.05), and Lasso regression. Additionally, Lasso coefficient paths and cross-validation curves were used to visualize the Lasso regression process, as shown in Fig. 3. From the results, we selected a feature set based on one standard error from the minimum lambda (lambda.min). This selection criterion reduced the number of variables, thereby enhancing the simplicity of the model. Three key radiomic features were identified.

### Model performance

**Fig. 4:**
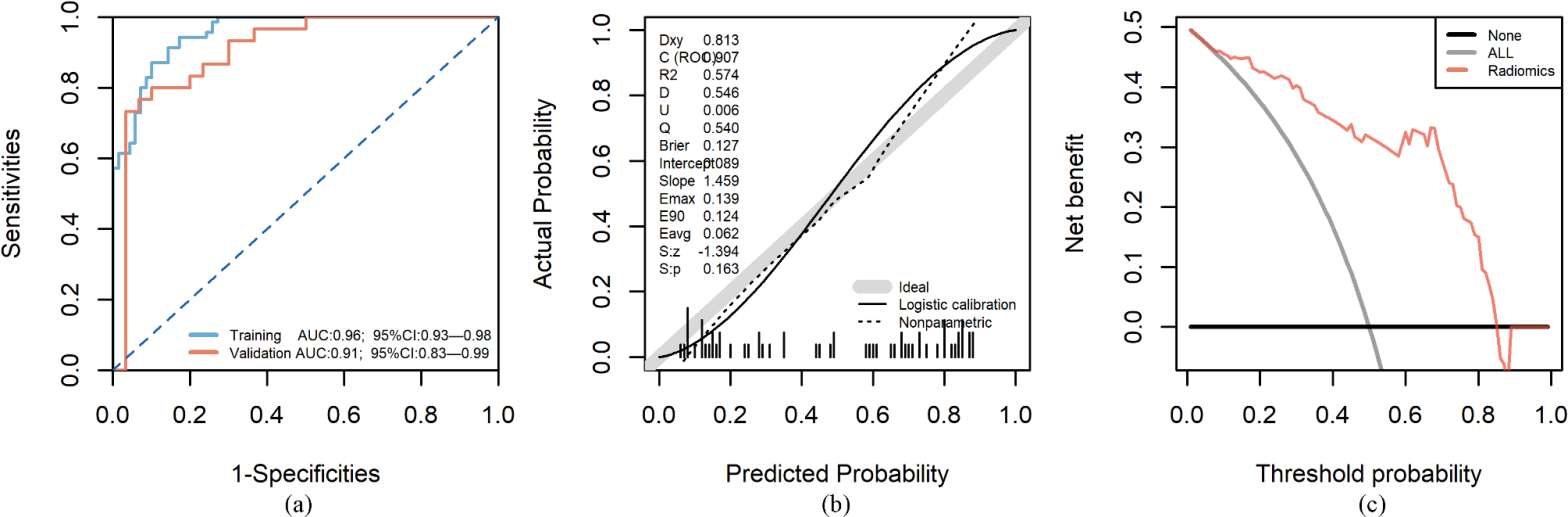
The ROC curve (**a**), calibration curve (**b**), and DCA decision curve (**c**) of the validation set.

The random forest (RF) model demonstrated excellent performance in both the training and validation cohorts. In the training cohort, the model achieved an AUC of 0.96 (95% CI 0.93–0.98) and an accuracy of 88%. In the validation cohort, the model’s AUC was 0.91 (95% CI 0.83–0.99), with an accuracy of 82% (Table 2). Calibration curves showed minimal deviation, with Spiegelhalter’s z-value of − 1.394 (*p* = 0.163), confirming reliable probability estimation (Fig. 4b). Furthermore, decision curve analysis (DCA) illustrated the model’s clinical utility across a broad range of threshold probabilities (0.1–0.8), as shown in Fig. 4c.

**Table 1:** Clinical data of the training set and validation set.

Characteristic	Training set		*P* ^1^	Validation set		*P* ^1^
	Normal *N* = 70	MS *N* = 70		Normal *N* = 30	MS *N* = 30	
Age, mean ± SD, years	36.64 ± 10.92	39.93 ± 8.83	0.052	37.53 ± 8.81	39.87 ± 9.55	0.329
Height, mean ± SD, cm	174.94 ± 6.63	173.53 ± 5.40	0.171	176.38 ± 5.98	175.13 ± 5.46	0.401
Weight, mean ± SD, kg	71.33 ± 7.47	87.04 ± 11.32	<0.001	71.63 ± 9.84	90.54 ± 11.45	<0.001
BMI, mean ± SD, kg.m^− 1^	23.30 ± 1.97	28.84 ± 2.95	<0.001	23.01 ± 2.79	29.49 ± 3.21	<0.001
Waistline (%)			<0.001			<0.001
< 90	70 (100.00%)	6 (8.57%)		30 (100.00%)	1 (3.33%)	
≥ 90	0 (0.00%)	64 (91.43%)		0 (0.00%)	29 (96.67%)	
Hyperglycemia (%)			<0.001			<0.001
No	70 (100.00%)	52 (74.29%)		30 (100.00%)	19 (63.33%)	
Yes	0 (0.00%)	18(25.71%)		0 (0.00%)	11(36.67%)	
Hypertension (%)			<0.001			<0.001
No	70 (100.00%)	13 (18.57%)		30 (100.00%)	5 (16.67%)	
Yes	0 (0.00%)	57 (81.43%)		0 (0.00%)	25 (83.33%)	
Fasting TG (%)			<0.001			<0.001
< 1.7	70 (100.00%)	7 (10.00%)		30 (100.00%)	5 (16.67%)	
≥ 1.7	0 (0.00%)	63 (90.00%)		0 (0.00%)	25 (83.33%)	
Fasting HDL-c (%)			<0.001			<0.001
≥ 1.04	70 (100.00%)	24 (34.29%)		30 (100.00%)	12 (40.00%)	
< 1.04	0 (0.00%)	46 (65.71%)		0 (0.00%)	18 (60.00%)	

^1^Independent Samples t-test; Pearson’s Chi-squared test.

*BMI* Body mass index, *MS* Metabolic syndrome, *HDL-c* High-density lipoprotein cholestero, *TG* Triglycerides.

**Table 2:** The performance of the infrared thermography-based radiomics model.

	Training set	Validation set
AUC	0.96 95%CI 0.93–0.98	0.91 95%CI 0.83–0.99
Accuracy	0.88	0.82
Sensitivity	0.91	0.80
Specificity	0.84	0.83

**Fig. 5:**
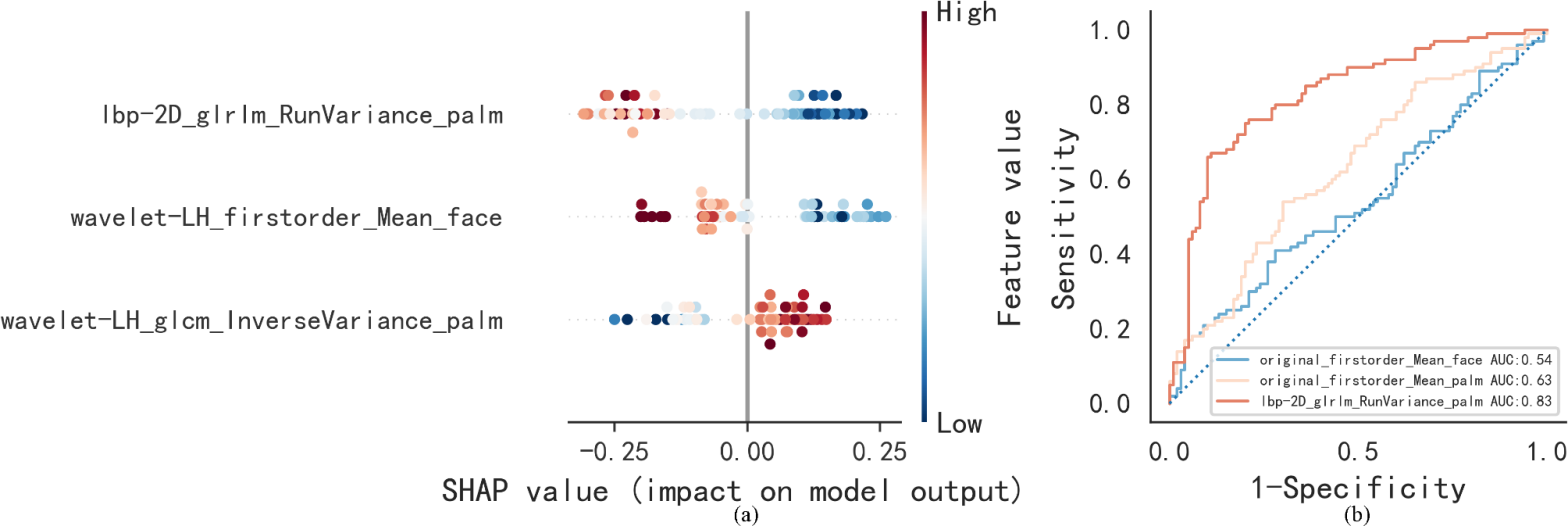
SHAP beeswarm plot: Interpreting feature influence in a random forest classifier (**a**), ROC curves of lbp-2D_glrlm_RunVariance_palm and one-dimensional statistical features in distinguishing MS (**b**).

Figure 5a presents a beeswarm plot of SHAP (Shapley Additive Explanation) values obtained from the radiomics random forest (RF) model. This plot visually represents the contribution of each feature to the classification decisions. SHAP values are a consistent and theoretically robust method for quantifying the influence of individual features on model predictions, illustrating how each feature either increases or decreases the model’s output^20,21^. In the plot, the horizontal axis represents the magnitude of the SHAP values, indicating the influence of each feature on the model’s predictions, while the vertical axis lists the features in order of their overall importance, from most to least significant.

### Thermal distribution patterns

Radiomic analysis revealed three robust biomarkers demonstrating superior diagnostic efficacy in differentiating metabolic syndrome patients from healthy controls (all AUC values calculated across the entire dataset), including lbp-2D_glrlm_RunVariance_palm (AUC: 0.83), wavelet-LH_firstorder_Mean_face (AUC: 0.74), and wavelet-LH_glcm_InverseVariance_palm (AUC: 0.70). These biomarkers significantly outperformed first-order temperature statistics, with traditional metrics showing limited discriminative ability (mean facial AUC: 0.63; palmar AUC: 0.54), as illustrated in Fig. 5b.

## Discussion

### Methodological and clinical advancements

Infrared thermography (IRT), as a functional imaging modality, offers a distinct advantage in capturing systemic metabolic changes through whole-body temperature distribution patterns. Unlike traditional structural imaging techniques (e.g., CT, MRI), which focus on morphological abnormalities, IRT visualizes dynamic thermal signatures generated by metabolic activity and vascular function. Our study utilizes this capability by analyzing high-dimensional radiomic features from facial and palmar regions, which are less prone to external interference and more reflective of systemic metabolic dysregulation.

The discriminative power of texture features, such as lbp-2D_glrlm_RunVariance, reflects spatial thermal heterogeneity caused by metabolic dysfunction, aligning with the pathophysiology of MS. Specifically, these features align with underlying mechanisms such as chronic inflammation-driven endothelial dysfunction^5–7^ and sympathetic hyperactivity in insulin resistance^8^. These underlying mechanisms alter microvascular perfusion and heat distribution, and radiomics can quantify with greater sensitivity than mean-temperature approaches.

Unlike structural imaging modalities such as CT and MRI, which are constrained by anatomical regions of interest (ROIs), IRT lacks standardized morphological references. As a result, shape features were excluded to mitigate segmentation-dependent biases, with emphasis instead placed on intensity- and texture-based biomarkers. This approach ensures that the analysis remains unbiased by anatomical variability, focusing instead on functional thermal patterns that reflect metabolic disturbances. Our multi-filter approach (LoG, LBP2D, wavelet decomposition) enhanced the detection of MS-associated thermal signatures, resulting in improved pattern discrimination compared to single-modality analyses. This methodology has proven to be more effective in extracting discriminative features, such as lbp-2D_glrlm_RunVariance_palm, and demonstrates the superiority of radiomics over traditional metrics (AUC: 0.83 vs. 0.63 for mean facial temperature).

The clinical applicability of IRT-based radiomics depends on the establishment of standardized protocols to mitigate device variability (e.g., temperature resolution, pixel density). Key procedural measures include^22^: Participants should refrain from consuming alcohol, caffeine, engaging in physical exercise, or being exposed to extreme temperatures for at least 4 h prior to imaging. Environmental Controls: A stable room temperature and humidity should be maintained, while minimizing airflow or any potential sources of radiation interference to ensure consistency in image acquisition. Image Acquisition: It is essential to maintain a fixed distance between the participant and the camera, ensuring precise alignment. The camera should be positioned perpendicularly to the regions of interest (ROIs), such as the facial and palmar areas, to avoid any distortions in the acquired data.

Post-acquisition image processing techniques further enhance robustness. For example, Shafiq-UI-Hassan et al.^23^ demonstrated that voxel resampling reduces feature size dependency, while normalization improves inter-device reproducibility. These steps, in conjunction with established frameworks such as the Glamorgan Protocol^24^ and the 2023 Chinese Medicine guidelines^25^, enable automated, reproducible workflows, which are essential for clinical adoption.

### Clinical implications

The clinical scalability of IRT-based radiomics is evident in its non-contact, rapid screening protocol, which can be completed in within 5 min. This makes it particularly valuable in resource-limited primary care settings, where advanced imaging modalities are often unavailable. Early detection of subclinical thermal anomalies—such as lbp-2D_glrlm_RunVariance—could facilitate lifestyle interventions to delay the onset of diabetes or cardiovascular complications.

Notably, our findings align with recent studies exploring IRT in metabolic disorders. For example, Thirunavukkarasu et al.^26^ achieved 94.28% accuracy in diabetes detection using tongue thermography, while Arteaga-Marrero et al.^13^ reported an AUC of 0.92 for diabetic foot ulcer screening. However, these studies focused on localized regions or single-disease endpoints. Our systemic approach, which combines facial and palmar biomarkers, provides a holistic view of metabolic dysregulation, bridging a critical gap in IRT research.

### Limitations and future directions

Despite promising results, several limitations warrant attention. First, the single-center retrospective design may introduce selection bias, as all participants were recruited from a physical examination cohort due to their inherent health awareness. Second, excluding female subjects (though justified by menstrual cycle-induced thermal variability^27^) limits generalizability. Future studies should specifically explore sex-specific radiomic signatures using phase-synchronized IRT acquisitions. Third, while the LASSO regression and SHAP analysis improved interpretability, the biological basis of selected features (e.g., lbp-2D_glrlm_RunVariance) remains unclear. Multimodal studies integrating IRT radiomics with vascular imaging (e.g., Doppler ultrasound) could elucidate underlying mechanisms. Lastly, external validation in diverse populations is essential before clinical translation.

High-dimensional thermal data embedded in IRT images—such as hundreds of pixels per ROI—provide a wealth of information that extends beyond manual interpretation. Traditional analyses, relying on mean or extreme temperature values, fail to capture complex spatial patterns such as texture variations enhanced by wavelet transforms or Local Binary Patterns (LBP2D). Our approach, integrating multiple filters, proved instrumental in enhancing the precision of feature extraction, thus revealing more subtle and complex thermal patterns indicative of metabolic dysfunction.

## Conclusion

This study establishes IRT-based radiomics as a transformative approach for metabolic syndrome (MS) screening. By integrating high-throughput radiomics with machine learning, we identified texture and filter-enhanced thermal biomarkers, such as lbp-2D_glrlm_RunVariance, which significantly outperformed traditional temperature metrics. Several key advancements were made in this research:

First, a standardized workflow was established by combining IBSI-compliant protocols with IRT-specific optimizations, such as environmental controls and multi-scale filtering, addressing historical inconsistencies in thermal imaging analysis. Second, our work enabled systemic biomarker discovery, with palmar features emerging as dominant indicators in SHAP rankings, pointing to microcirculatory dysfunction as an early mechanism in MS, detectable even before structural pathology. Moreover, the clinical scalability of this approach was demonstrated with a sub-5-minute, non-contact screening protocol, making MS screening accessible, especially in primary care settings where advanced imaging may be unavailable.

While the study is limited by retrospective, single-center data and an male-only cohort cohort, it successfully bridges functional IRT with precision radiomics, positioning thermal imaging as a complementary tool to CT/MRI in metabolic phenotyping. Future research should focus on validation in diverse populations, sex-stratified models, and the application of real-time IRT radiomics for monitoring therapeutic interventions.

## Data Availability

The data that support the findings of this study are available on request from the corresponding author. The data are not publicly available due to privacy or ethical restrictions.

## Abbreviations

AUC: Area under the curve
CT: Computed tomography
DCA: Decision curve analysis
IRT: infrared thermography
MRI: Magnetic resonance imaging
MS: Metabolic syndrome
LASSO: Least absolute shrinkage and selection operator
PCC: Pearson correlation coefficients
PET: Positron emission tomography
RF: Random forest
Shap: Shapley additive explanations

## References

[1] Lambin, P. et al. Radiomics: extracting more information from medical images using advanced feature analysis. *Eur. J. Cancer*. **48**, 441–446 (2012). 22257792 10.1016/j.ejca.2011.11.036 PMC4533986

[2] Kumar, V. et al. Radiomics: the process and the challenges. *Magn. Reson. Imaging*. **30**, 1234–1248 (2012). 22898692 10.1016/j.mri.2012.06.010 PMC3563280

[3] Qiao, M., Hu, Y., Guo, Y., Wang, Y. & Yu, J. Breast tumor classification based on a computerized breast imaging reporting and data system feature system. *J. Ultrasound Med.* **37**, 403–415 (2018). 28804937 10.1002/jum.14350

[4] Pennes, H. H. Analysis of tissue and arterial blood temperatures in the resting human forearm. *J. Appl. Physiol.* **85**, 5–34 (1998). 9714612 10.1152/jappl.1998.85.1.5

[5] Hotamisligil, G. S. Inflammation and metabolic disorders. *Nature*. **444**, 860–867 (2006). 17167474 10.1038/nature05485

[6] Saltiel, A. R. & Olefsky, J. M. Inflammatory mechanisms linking obesity and metabolic disease. *J. Clin. Investig.* **127**, 1–4 (2017). 28045402 10.1172/JCI92035 PMC5199709

[7] Grandl, G. & Wolfrum, C. Hemostasis, endothelial stress, inflammation, and the metabolic syndrome. *Semin. Immunopathol.* **40**, 215–224 (2018). 29209827 10.1007/s00281-017-0666-5 PMC5809518

[8] Geerling, J. J. et al. Sympathetic nervous system control of triglyceride metabolism: novel concepts derived from recent studies. *J. Lipid Res.* **55**, 180–189 (2014). 24285857 10.1194/jlr.R045013 PMC3886657

[9] Singh, D. & Singh, A. K. Role of image thermography in early breast cancer detection—past, present and future. *Comput. Methods Progr. Biomed.* **183**, 105074 (2020). 10.1016/j.cmpb.2019.105074 31525547

[10] De Camargo, V. M. B., Ulbricht, L., Coninck, J. C. P., Ripka, W. L. & Gamba, H. R. Thermography as an aid for the complementary diagnosis of nodules in the thyroid gland. *BioMed. Eng. OnLine*. **21**, 41 (2022). 35761269 10.1186/s12938-022-01009-3 PMC9235134

[11] Collins, A. J., Ring, E. F., Cosh, J. A. & Bacon, P. A. Quantitation of thermography in arthritis using multi-isothermal analysis. I. The thermographic index. *Ann. Rheum. Dis.* **33**, 113–115 (1974). 4821383 10.1136/ard.33.2.113 PMC1006220

[12] Schlager, O. et al. Correlation of infrared thermography and skin perfusion in Raynaud patients and in healthy controls. *Microvasc. Res.* **80**, 54–57 (2010). 20144625 10.1016/j.mvr.2010.01.010

[13] Arteaga-Marrero, N., Hernández-Guedes, A., Ortega-Rodríguez, J. & Ruiz-Alzola, J. State-of-the-art features for early-stage detection of diabetic foot ulcers based on thermograms. *Biomedicines* **11**, 3209 (2023). 38137430 10.3390/biomedicines11123209 PMC10741214

[14] Zhang, W. et al. Preliminary study of finger temperature recovery in patients with diabetes mellitus following cold stimulation. *Diabetes Metab. Res.* **40**, e3706 (2024). 10.1002/dmrr.3706 37545385

[15] Gao, M. J. et al. A preliminary study on infrared thermograph of metabolic syndrome. *Front. Endocrinol. (Lausanne)*. **13**, 851369 (2022). 35498430 10.3389/fendo.2022.851369 PMC9042650

[16] Thiruvengadam, J. & Mariamichael, A. A preliminary study for the assessment of hypertension using static and dynamic IR thermograms. *Biomedical Eng./Biomedizinische Technik*. **63**, 197–206 (2018). 10.1515/bmt-2016-0237 28675748

[17] Thiruvengadam, J., Anburajan, M., Menaka, M. & Venkatraman, B. Potential of thermal imaging as a tool for prediction of cardiovascular disease. *J. Med. Phys.* **39**, 98–105 (2014). 24872607 10.4103/0971-6203.131283 PMC4035622

[18] Chinese Elderly Type 2 Diabetes Prevention and Treatment of Clinical Guidelines Writing Group, Geriatric Endocrinology and Metabolism Branch of Chinese Geriatric Society, Geriatric Endocrinology and Metabolism Branch of Chinese Geriatric Health Care Society, Geriatric Professional Committee of Beijing Medical Award Foundation, & National Clinical Medical Research Center for Geriatric Diseases (PLA General Hospital). [Clinical guidelines for prevention and treatment of type 2 diabetes mellitus in the elderly in China (2022 edition)]. *Zhonghua Nei Ke Za Zhi*. **61**, 12–50 (2022). 34979769 10.3760/cma.j.cn112138-20211027-00751

[19] Zwanenburg, A. et al. The image biomarker standardization initiative: standardized quantitative radiomics for high-throughput image-based phenotyping. *Radiology*. **295**, 328–338 (2020). 32154773 10.1148/radiol.2020191145 PMC7193906

[20] Lundberg, S. & Lee, S. I. A unified approach to interpreting model predictions. http://arxiv.org/abs/1705.07874 (2017).

[21] Wang, J., Wiens, J. & Lundberg, S. Shapley flow: A graph-based approach to interpreting model predictions. http://arxiv.org/abs/2010.14592 (2021).

[22] Moreira, D. G. et al. Thermographic imaging in sports and exercise medicine: A Delphi study and consensus statement on the measurement of human skin temperature. *J. Therm. Biol*. **69**, 155–162 (2017). 29037377 10.1016/j.jtherbio.2017.07.006

[23] Shafiq-ul-Hassan, M. et al. Voxel size and Gray level normalization of CT radiomic features in lung cancer. *Sci. Rep.* **8**, 10545 (2018). 30002441 10.1038/s41598-018-28895-9 PMC6043486

[24] Ammer, K. The Glamorgan Protocol for recording and evaluation of thermal images of the human body. (2008).

[25] China Association of Chinese Medicin. *Standard of Infrared Image Acquisition and Image Analysis of Traditional Chinese Medicine* (Standards Press of China, 2023).

[26] Thirunavukkarasu, U., Umapathy, S., Krishnan, P. T. & Janardanan, K. Human tongue thermography could be a prognostic tool for prescreening the Type II diabetes mellitus. *Evid.-Based Complement. Altern. Med.* **2020**, 1–16 (2020). 10.1155/2020/3186208 PMC7201785 32419801

[27] Fuller-Jackson, J. P., Dordevic, A. L., Clarke, I. J. & Henry, B. A. Effect of sex and sex steroids on brown adipose tissue heat production in humans. *Eur. J. Endocrinol.* **183**, 343–355 (2020). 32508310 10.1530/EJE-20-0184

